# Autistic traits are associated with the functional connectivity of between—but not within—attention systems in the general population

**DOI:** 10.1186/s12868-020-00603-2

**Published:** 2020-11-23

**Authors:** Sayaka Yoshimura, Kei Kobayashi, Tsukasa Ueno, Takashi Miyagi, Naoya Oishi, Toshiya Murai, Hironobu Fujiwara

**Affiliations:** 1 Faculty of Human Health Science, Graduate School of Medicine, Kyoto University, 53 Shogoin-Kawaharacho, Sakyo-Ku, Kyoto 606-8507 Japan; 2 Department of Neuropsychiatry, Graduate School of Medicine, Kyoto University, 54 Shogoin-Kawaharacho, Sakyo-Ku, Kyoto 606-8507 Japan; 3 Medical Innovation Center, Graduate School of Medicine, Kyoto University, 54 Shogoin-Kawaharacho, Sakyo-Ku, Kyoto 606-8507 Japan; 4 RIKEN Center for Advanced Intelligence Project, Artificial Intelligence Ethics and Society Team, Nihonbashi 1-chome Mitsui Building, 15th floor, 1-4-1 Nihonbashi, Chuo-Ku, Tokyo 103-0027 Japan

**Keywords:** Attention, Attention network, Autistic traits, Resting-state functional magnetic resonance imaging, Functional connectivity

## Abstract

**Background:**

Previous studies have demonstrated that individuals with autism spectrum disorder (ASD) exhibit dysfunction in the three attention systems (*i.e.*, alerting, orienting, and executive control) as well as atypical relationships among these systems. Additionally, other studies have reported that individuals with subclinical but high levels of autistic traits show similar attentional tendencies to those observed in ASD. Based on these findings, it was hypothesized that autistic traits would affect the functions and relationships of the three attention systems in a general population. Resting-state functional magnetic resonance imaging (fMRI) was performed in 119 healthy adults to investigate relationships between autistic traits and within- and between-system functional connectivity (FC) among the three attention systems. Twenty-six regions of interest that were defined as components of the three attention systems by a previous task-based fMRI study were examined in terms of within- and between-system FC. We assessed autistic traits using the Autism-Spectrum Quotient.

**Results:**

Correlational analyses revealed that autistic traits were significantly correlated with between-system FC, but not with within-system FC.

**Conclusions:**

Our results imply that a high autistic trait level, even when subclinical, is associated with the way the three attention systems interact.

## Background

When the process of attention is applied to manage competing environmental information, the result is bias selection and action toward one choice while separating out interference from the remaining information [3]. Thus, attention is a primary component of cognition that allows humans to experience the world and that influences perception, memory, behavior and, possibly, the direction of brain development.

Attention is thought to be composed of three anatomically and functionally independent systems: the alerting system (alerting), the orienting system (orienting), and the executive control system (EC; for a review, see [54]). Alerting is the most elementary aspect of attention and is the system responsible for achieving and maintaining a state of sensitivity to incoming information. Orienting is associated with the ability to assign priority to sensory inputs by selecting a location or sensory modality [49]. EC is a multidimensional and relatively complex system that includes mental operations, such as divided attention and detecting and resolving conflicts, that are responsible for controlling behaviors and thoughts. These three systems work more efficiently when interacting with each other [13, 24, 67].

Previous studies have reported that individuals with autism spectrum disorder (ASD) exhibit attentional dysfunction in all three systems (for a review, see [36]). ASD is a neurodevelopmental disorder characterized by deficits in social interaction and the presence of repetitive and restricted behaviors [2]. From early childhood, individuals with ASD have pervasive abnormalities in attention [1] that could influence the development of the two aforementioned diagnostic features of ASD. In particular, impairments in orienting have been consistently reported in behavioral and imaging studies [6, 36, 37].

Furthermore, other behavioral and imaging studies have reported that the relationships among the three attention systems in individuals with ASD are different to those in neurotypical individuals. For example, a behavioral study found that the pattern of the relationship between alerting and EC in adolescents with ASD differs from that in typically developing adolescents [35]. Similarly, a resting-state fMRI study revealed differences in the temporal dynamics from orienting to EC between ASD and typically developing individuals [10].

It is also possible that the presence of autistic traits in people in the general population may affect attention, including functional relationships among the three attention systems in which there are strong individual differences (*e.g.*, [22]). Autistic traits are continuously distributed throughout the general population and range from individuals with almost no autistic traits to severely impaired and diagnosed individuals (*e.g.*, [14]). Several behavioral studies have reported that individuals with subclinical but high levels of autistic traits exhibit attentional tendencies that are similar to those observed in ASD when compared to individuals with fewer autistic features (*e.g.*, [47, 69]).

Additionally, imaging studies have demonstrated that autistic traits are dimensionally related to functional connectivity (FC) in neurotypical individuals. For example, FC of the social processing network is negatively related to autistic traits in typically developing adults [18]. Another study found that whole-brain FC is highest in controls, intermediate in the unaffected siblings of individuals with ASD, and lowest in individuals with ASD [44]. Taken together, these findings indicate that autistic traits in the general population might be associated with FC between regions that are responsible for the same (*i.e.*, within-system) or two different (*i.e.*, between-system) attention systems. However, to the best of our knowledge, no studies have examined these relationships in the general population.

Thus, the primary aim of the present study was to investigate the relationship between autistic traits in a general population and within-and between-system FC among brain areas that have been reported as components of the alerting, orienting, and EC networks [23].

## Results

### Psychological data

**Fig. 1:**
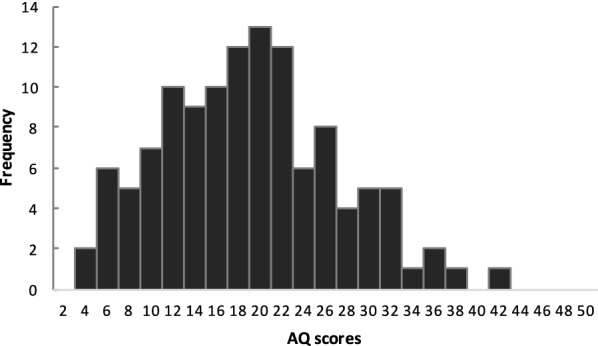
Distribution of the AQ scores

The mean ± standard deviation (*SD*) of the Autism-Spectrum Quotient (AQ) scores in the present study was 18.4 ± 8.0, which was identical to the value observed in a study of the general population assessed using the Japanese version of the AQ [64]. Kolmogorov–Smirnov tests revealed that the AQ scores had normal distributions (*p* = 0.20; Fig. 1).

### Relationships between AQ scores and FC

**Fig. 2:**
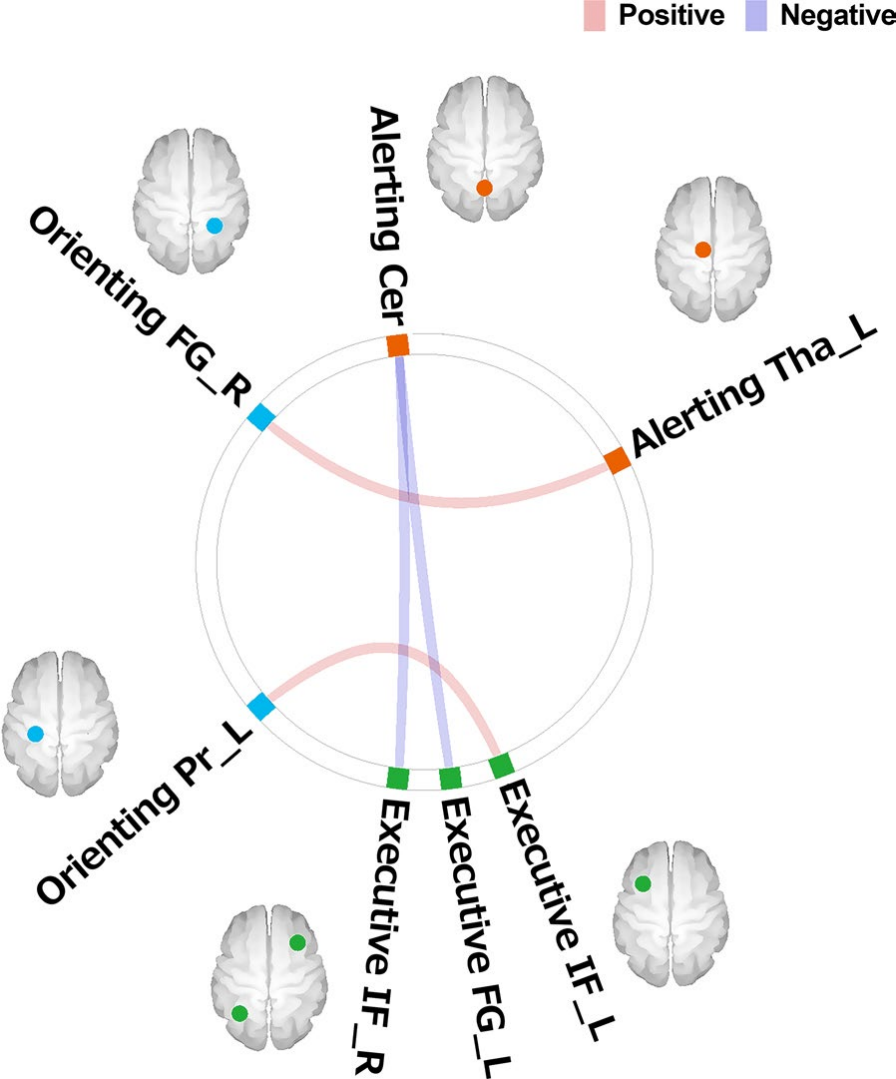
Brain regions showing significant associations between the AQ score and FC value. Tha = Thalamus; Cer = Cerebellar vermis; FG = Fusiform gyrus; Pr = Precentral gyrus; IF = Inferior frontal gyrus; L = left; R = right

Correlation analyses revealed significant relationships between the AQ scores and the between-system FC values (Table 1 and Fig. 2). In contrast, there were no significant associations between the AQ scores and the within-system FC values. To investigate gender differences in the relationships between autistic traits and the FC values, we assessed possible correlations between the AQ scores and the within- and between FC values with age as the only covariate. The results were essentially the same as those obtained after using both age and gender as covariates (Additional file 1: Table S1).

**Table 1:** The two ROIs that exhibited significant relationships between AQ scores and FC values

	T-value	FDR adjusted *p*-value	*r*
Alerting-Orienting			
L. thalamus-R. fusiform gyrus	3.42	0.021	0.30
Alerting-Executive control			
Cerebeller vermis-L. fusiform gyrus	− 3.37	0.026	0.30
Cerebeller vermis-R. inferior frontal gyrus	− 3.48	0.013	0.31
Orienting-Executive control			
L. precentral gyrus-L. inferior frontal gyrus	3.46	0.019	0.31

L = Left; R = Right; AQ = Autism-spectrum quotient; ROI = Regions of interests

## Discussion

The primary goal of the present study was to examine whether autistic traits in a general population would be associated with within- and/or between-system FC among the three attentional systems, *i.e.*, the alerting, orienting, and EC systems. There were significant associations between AQ scores and between-system FC values but not between AQ scores and within-system FC values; the AQ scores had positive relationships with FC between the regions responsible for alerting and orienting and with FC between the regions responsible for orienting and EC, but a negative relationship with FC between the regions responsible for alerting and EC.

In the present study, a greater degree of autistic tendencies was related to stronger FC between the thalamus, which is the center of alerting, and the fusiform gyrus responsible for orienting (*i.e.*, the fusiform face area [FFA]). Previous studies have consistently reported that orienting is impaired in individuals with ASD (for a review, see [36]) and that this function is weaker in individuals with a high level of autistic traits from a general population [47, 69]. Alerting, or the function of achieving and maintaining a state of sensitivity to incoming information, promotes orienting [13, 28, 59]. Thus, the present findings that there was stronger FC between alerting and orienting in individuals with higher levels of autistic traits suggest that the weaker function in orienting might be compensated for by alerting.

Based on evidence showing that the FFA is an important factor in those with ASD and asymptomatic individuals with higher levels of autistic traits, the notion that this region presents a weaker function in orienting is reasonable. The FFA is known to be specialized for face perception (*e.g.*, [34]). Individuals with ASD exhibit impairments in face perception (for a review, see [65]) and a functionally and structurally atypical FFA [20, 50, 57, 63]. Additionally, a positron emission tomography (PET) scan study revealed that cholinergic deficits in the fusiform gyrus, which are related to impairments in social interaction, are evident in subjects with ASD [60]. Because the orienting system, but not the other attention systems, is cholinergic [49], these findings suggest that orienting deficits in individuals with ASD might be partly due to differences in the FFA and that higher levels of autistic traits might be associated with more deficient orienting. Because individuals with subclinical but high levels of autistic traits also exhibit diminished activation in the fusiform gyrus during a face recognition task [16], people with higher levels of autistic traits might have a weaker orienting function that is related to alterations in the FFA.

The weaker orienting function, associated with autistic traits, could also have impacted FC between orienting and EC. The present results revealed that a greater degree of autistic tendencies was related to stronger connectivity between the precentral gyrus (orienting) and the inferior frontal gyrus (IFG; EC). The function of the precentral gyrus in orienting is thought to be similar to that of the frontal eye field [23], which implements eye movements such as saccades. Saccades are the phenomenon of initiating rapid ballistic shifts in eye gaze that are needed for attentional shifts during the orienting process [5]. Several studies have reported that saccades are impaired in individuals with ASD (for a review, see [43]). Moreover, because saccades are also impaired in unaffected first-degree relatives of individuals with ASD [42], those with a greater degree of autistic traits might have weaker saccade function. On the other hand, Kane et al. [33] found that people with higher levels of executive function perform better on a saccade task than those with lower levels of executive function, which suggests that EC improves the function of saccades. Taken together with functions related to the IFG, which involves the selection of information to adjust sensory inputs [7, 68], the present results suggest that weaker saccade function in individuals with higher levels of autistic traits might be compensated for by EC.

In the present study, a greater degree of autistic tendencies was also related to weaker FC between the cerebellar vermis (alerting) and the two EC regions, *i.e.*, the fusiform gyrus (as mentioned in the Methods section, this region is regarded as a non-face area) and the IFG. This finding may indicate that there is a delayed development of the alerting-EC relationship in individuals with a greater degree of autistic tendencies. In general, relative to alerting and orienting, EC follows a protracted development period that can persist through adolescence (for reviews, see [11, 19]). During that developmental course, while functional interactions among the attentional systems (except the alerting and EC interactions) are characterized in childhood and persist into adulthood, the direction of the alerting-EC interaction is generally reversed by 12 years of age such that the positive association in childhood changes into a negative one during adolescence [45, 53]. On the other hand, a behavioral study examining the interactions among the attention systems found that there is a positive association between alerting and EC in adolescents with ASD [35] whereas typically developing adolescents have a negative association [27] or lack an association [35]. Considering that adults with ASD do not exhibit an alerting-EC association [25] and that EC skills mature at a slower pace in ASD individuals than typically developing people (for a review, see [17]), the findings in previous behavioral studies suggest that the functional reversal of the alerting-EC association may be delayed in ASD and that the development of this association in adults with ASD may be in a stage equivalent to that observed in typically developing adolescents. Moreover, together with the idea that age-related changes in the interactions of attentional behaviors are associated with changes in FC [55] and findings about age being an important factor to consider when assessing FC alterations in ASD [32, 46], it is likely that individuals with ASD show delayed or different patterns of developmental changes in FC between alerting and EC. The present results showing that higher levels of autistic tendencies were related to weaker FC between alerting and EC may indicate that this type of atypical development also occurs in non-clinical individuals with high levels of autistic traits.

The present findings also have clinical implications regarding the mental health of non-clinical individuals with higher levels of autistic traits. A considerable number of studies have documented higher rates of psychiatric problems, such as anxiety and depression, in this non-clinical population (*e.g.*, [41, 51, 66]); these issues could be derived, at least in part, from orienting dysfunction. In other words, these disorders could be due to attention biases, such as reduced attention to positive information, excessive attention to negative information, and local processing biases; such kinds of biases could induce a distressed mood [4, 12, 29, 58]. Because this type of orienting dysfunction has been reported in non-clinical individuals with higher levels of autistic traits [39, 56], it may be a cause of the elevated rates of other psychiatric conditions in this population. Moreover, if alerting and EC compensate for orienting in this population, as suggested by the present findings, then training alerting and EC functions might result in maintenance of mental health through functional improvements in orienting. The function of attention networks, especially that of EC, can be improved by changes in brain states induced by exercise [21, 31] or meditation [61]. Thus, investigations on the association between training attentional functions (through these activities) and mental health in this population would likely to provide interesting results.

The present study has several limitations that should be noted. First, the present findings are limited by the age of the participants. As described above, it is possible that the directions of the interactions among the attention systems might be partly reversed with increasing age. Future research using elderly populations will clarify whether this functional reverse might happen and/or when it does. Second, the relationships between actual attentional functions and the between-system FC of attention networks were not assessed in the present study. Although resting-state brain networks resemble task-evoked networks [62], the relationships among the three attention systems (*i.e.*, between-system FC) might change during active attention. Therefore, task-dependent FC should be studied using a combination of imaging and behavioral data to reveal possible changes in the relationships among active attention systems and in relation to the presence of compensatory mechanisms. Third, future investigations should consider within-system FC. In the present study, autistic traits were not associated with within-system FC whereas stronger and weaker FC within orienting have been reported in individuals with ASD [26, 37]. It is possible that the present results were influenced by the non-clinical nature of the participants or the relatively small sample size. Thus, studies with larger sample sizes should be conducted to determine whether autistic traits in the general population are associated with within-system FC.

## Conclusion

This study demonstrated that autistic traits in the general population were associated with between-system FC among the neural substrates of the three attention systems. These results suggest that autistic traits affect relationships among the three systems and could possibly induce changes in the efficiency of attention. Although further research is necessary, the present study provided novel insights into the individual differences associated with attentional functions.

## Methods

### Participants

This study was conducted as part of a project investigating the association between personalized values and lifestyle habits. The participants included 119 healthy volunteers (45 females, mean age ± *SD*: 36.2 ± 14.3 years) who were confirmed as right-handed using the Edinburgh Handedness Inventory [48]. Interviews conducted by two well-trained psychiatrists confirmed that none of the participants had any type of psychiatric disorder, severe medical or neurological illness, or severe head injury. The IQ scores of the participants were estimated with the Japanese version of the Adult Reading Test (JART; [40]); all participants fell within the normal range (full-scale IQ, *M* = 109.5, *SD* = 6.4; verbal IQ, *M* = 111.0, *SD* = 7.4; and performance IQ, *M* = 106.3, *SD* = 4.9). After having the experimental procedures fully explained, all participants provided written informed consent prior to participation in the study.

### Psychological questionnaire

The AQ is a self-report and one of the most commonly used questionnaires assessing the degree of autistic traits in an individual [8]. Its reliability and validity have been tested and confirmed in many studies from various countries (*e.g.*, [8, 38, 64]). The measure consists of 50 items that are each scored as 1 point if the respondent records mild or strong abnormal or autistic-like behaviors; thus, a higher AQ score is indicative of a higher degree of autistic traits. We used the Japanese version of the AQ [64] in this study.

### MRI acquisition

Image scanning was conducted with a single-shot gradient echo planar imaging (EPI) pulse sequence on a 3-T MRI unit (Tim-Trio, Siemens, Erlangen, Germany) with a 40-mT/m gradient, a receiver-only 32-channel phased-array head coil, and small elastic pads placed on both sides of the head to minimize head motion. Structural MRI data were acquired using three-dimensional magnetization-prepared rapid gradient echo (3D-MPRAGE) sequences with the following parameters: repetition time (TR), 2000 ms; echo time (TE), 3.4 ms; inversion time, 990 ms; field of view (FOV), 225 × 240 mm; matrix size, 240 × 256; resolution, 0.9375 × 0.9375 × 1.0 mm^3^; and 208 total axial sections without intersection gaps. We instructed the participants to visually concentrate on a fixation cross in the center of the screen and to avoid thinking about anything specific. Functional images were obtained during a single 10-min session, while the subjects kept their eyes open, and using a sequence with the following characteristics: TR, 2500 ms; TE, 30 ms; flip angle, 80°; FOV, 212 × 212 mm; matrix size, 64 × 64; in-plane spatial resolution, 3.3125 × 3.3125 mm^2^; 40 total axial slices; and slice thickness, 3.2 mm with 0.8 mm gaps in ascending order. A dual-echo gradient echo dataset for B0 field mapping was also acquired for distortion correction purposes. After the scanning, the subjects were asked questions about sleepiness during data acquisition. We recorded only answers denying sleep (no “fell asleep” or “was almost asleep” replies).

### Image processing

The resting state-fMRI dataset was corrected for EPI distortions in the FSL software package (FMRIB’s software library ver. 5.0.9; http://www.fmrib.ox.ac.uk/fsl) using FMRIB’s Utility for Geometrically Unwarping EPIs (FUGUE), which unwarps EPI images based on fieldmap data. Subsequently, artifact components and motion-related fluctuations were removed from the images using FMRIB’s independent component analysis-based X-noiseifier [30].

We performed all imaging and statistical analyses using the CONN-fMRI Functional Connectivity toolbox (ver.17e; www.nitrc.org/projects/conn) with the statistical parametric mapping package (SPM12; http://www.fil.ion.ucl.ac.uk/spm). First, all functional images were realigned and unwarped, slice-timing corrected, co-registered with structural data, spatially normalized into the standard MNI space (Montreal Neurological Institute, Montreal, QC, Canada), outlier-detected (ART-based scrubbing), and smoothed using a Gaussian kernel of 8 mm full width at half maximum.

All preprocessing steps were performed using a default preprocessing pipeline for volume-based analysis (to MNI space). We segmented structural data into gray matter, white matter, and cerebrospinal fluid (CSF), and then normalized them in the same default preprocessing pipeline. The principal components of the signals from white matter and CSF and the translational and rotational movement parameters (with six other parameters representing their first-order temporal derivatives) were removed with a covariate regression analysis conducted using CONN. Using the implemented CompCor strategy [9], we reduced the effects of nuisance covariates, including fluctuations in resting-state fMRI signals from white matter, CSF, and their derivatives as well as realignment parameter noise. As recommended, band-pass filtering was performed with a frequency window of 0.008–0.09 Hz,this preprocessing step was found to increase the retest reliability.

### FC analysis

We conducted a region of interest (ROI)-to-ROI FC analysis in the present study. We specified 26 spherical clusters with 10-mm diameters and peak coordinates, as described in Fan et al. [23]. These authors conducted a task-based fMRI study that identified these regions as components of the three attention systems. Table 2 shows the ROIs in each system (coordinates are quoted in the Talairach space). Although each of the three systems includes the fusiform gyrus, the regions responsible for orienting were distinguished as the FFA and were differentiated from the regions responsible for alerting and EC. The alerting and EC regions were located outside of the boundary of the FFA: the right FFA had an average size of 1 cm^3^ and was located at the Talairach coordinates 40x, − 55y, and − 10z; the left FFA had an average size of 0.5 cm^3^ and was located at the Talairach coordinates − 35x, − 63y, and − 10z [34].

**Table 2:** Brain regions supporting the three attentional networks

Network	Brain region	BA	Coordinates		
			*x*	*y*	*z*
Alerting	R. superior temporal gyrus^a^	22	61	− 40	11
	Superior colliculus		6	− 28	− 7
	L. Thalamus		− 12	− 17	6
	R. thalamus		13	− 9	7
	L. inferior parietal lobe	40	− 50	− 20	21
	L. fusiform gyrus	37	− 42	− 62	0
	L. inferior frontal gyrus	47	− 32	27	0
	Cerebellar vermis		0	− 65	− 10
	L. superior parietal lobe^b^	7	− 36	− 46	50
Orienting	L. fusiform gyrus	37	− 34	− 60	− 5
	R. fusiform gyrus	37	30	− 47	− 6
	L. precentral gyrus^c^	6	− 38	− 8	41
	R. superior parietal lobe^d^	7	32	− 41	30
	L. superior frontal gyrus	6	− 10	7	57
	L. superior parietal lobe^e^	7	− 28	− 72	28
	R. postcentral gyrus	2	57	− 21	43
	L. precentral gyrus	4	− 30	− 26	53
Executive control	Thalamus^f^		− 22	− 27	3
	L. superior frontal gyrus^c^	6	− 16	4	44
	R. inferior frontal gyrus	45	36	26	15
	L. fusiform gyrus	37	− 36	− 60	1
	L. inferior frontal gyrus	47	− 34	20	5
	Cerebellar vermis		0	− 62	− 32
	R. middle frontal gyrus	6	36	− 5	50
	R. fusiform gyrus	37	44	− 58	1
	R. anterior cingulate gyrus	32	6	36	26

L = Left; R = Right; BA = Brodmann's area

^a^Subregion of temporal parietal junction

^b^Anterior intraparietal sulcus

^c^Close to frontal eye field

^d^Anterior intraparietal sulcus

^e^Junction of intraparietal and transverse occipital sulcus

The regions responsible for alerting included the thalamus, parietal regions (*e.g.*, temporoparietal junction), and the mid-frontal gyrus. The regions responsible for orienting included the superior parietal cortex and the frontal eye field. The orienting regions are roughly equivalent to the dorsal attention network in large-scale brain networks [15]. The EC regions included the anterior cingulate cortex and the dorsolateral prefrontal cortex, which overlaps with the front-parietal network and with the salience network in large-scale brain networks [52].

### Statistical analysis

We assessed the associations between the AQ scores and the within-and between-system FC values of the two ROIs using *t*-statistics by CONN with age and gender as covariates; we considered all false discovery rate-corrected *p*-values < 0.05 as statistically significant.

## Supplementary information

**Additional file 1: Table S1. **The two ROIs that exhibited significant relationships between AQ scores and FC values.

## Data Availability

The datasets generated and/or analyzed during the current study are not publicly available because we did not obtain the consent of participants to provide the datasets to third parties.
