# Supplementary material for: Autistic traits are associated with the functional connectivity of between—but not within—attention systems in the general population
Source: BMC Neurosci. 2020 Nov 23;21:49. doi: 10.1186/s12868-020-00603-2 (PMC7686764; doi:10.1186/s12868-020-00603-2)
Supplement: Supplementary file 1 — Additional file 1: Table S1. The two ROIs that exhibited significant relationships between AQ scores and FC values. [file 12868_2020_603_MOESM1_ESM.docx]

**Additional file 1: Table S1**. The two ROIs that exhibited significant relationships between AQ scores and FC values.
